# Pars Plana Vitrectomy with Internal Limiting Membrane Peeling in Traumatic Macular Hole: 14% Perfluoropropane (C_3_F_8_) versus Silicone Oil Tamponade

**DOI:** 10.1155/2017/3917696

**Published:** 2017-07-24

**Authors:** Ashraf Bor'i, Mahmoud A. Al-Aswad, Ahmed Abdelwahab Saad, Dina Hamada, Ashraf Mahrous

**Affiliations:** Ophthalmology Department, Zagazig University, Zagazig, Egypt

## Abstract

**Purpose:**

To evaluate the outcome of 23 G PPV and ILM peeling with 14% C_3_F_8_ compared with silicone oil tamponade in cases of TMHs without spontaneous closure.

**Methods:**

A retrospective comparative study included 33 eyes with TMHs; 7 eyes healed spontaneously, and the remaining 26 eyes have been treated with PPV and ILM peeling. Silicone oil was used as a tamponade for children or adults who refused to adopt face-down position (10 cases). In all other cases (16 cases), 14% C_3_F_8_ was used. These cases were followed up for 6 months postoperatively.

**Results:**

26 cases (22 males and 4 females) were reviewed, including 10 cases treated with silicone oil and 16 cases treated with 14% C_3_F_8_. Patients' age ranged from 9 to 54 years. The success rate was 90% in the silicone-filled (9/10) and 94% in the gas-filled (15/16) eyes. At 6 months, the mean BCVA was 0.3 ± 0.25 in the silicone group and 0.2 ± 0.13 in the gas group (*p* < 0.05).

**Conclusions:**

Cases of TMHs should be observed for spontaneous closure. PPV with ILM peeling should be conducted for nonclosing cases. Gas and silicone oil tamponades are equally successful in anatomical and visual outcomes. This trial is registered with CTRI/2017/06/008765.

## 1. Introduction

Macular holes, which are full-thickness defects that disrupt the foveal contour, are commonly idiopathic or age-related, but they may be traumatic due to blunt injury to the globe and are usually associated with localised or diffuse retinal edema, vitreous haemorrhages, retinal breaks and disinsertion, and choroidal rupture [1, 2].

Verifiably, Knapp [3] published the first case study of TMH in a patient with blunt eye trauma in 1869. Noyes [4] was the first to discover that TMH was a full-thickness defect inside the centre of the macula. The incidence of TMH varies from 1 to 9% between different studies and is more common in young male population [5–8].

The mechanism that derives the formation of TMH has remained a controversial subject. There are numerous speculations regarding the pathogenesis of TMH [8–12]. In the mid-1900s, one of the most widely accepted hypotheses stated that TMH potentially arose from retinal stretching caused by deformation during the trauma and/or the direct impact of the trauma on the posterior pole [8, 9]. Today, TMH is thought to be created not only by direct concussion of the globe but also by vitreous traction [13, 14]. Clinicians should observe patients with TMH for 4–6 months rather than attempt to surgically repair the injury, which has been advocated because of the possibility of spontaneous closure [15–19]. In this study, we evaluated the outcome of 23 G pars plana vitrectomy (PPV) and ILM peeling with 14% C_3_F_8_ tamponade versus silicone oil tamponade in cases of TMH without spontaneous closure.

## 2. Patients and Methods

Retrospective comparative study was done from May 2014 to September 2016. Thirty-three eyes of 33 patients (29 males and 4 females) with TMH were operated upon during this period. Eyes with submacular haemorrage, choroidal rupture, and rhegmatogenous retinal detachment were excluded from the study. All cases were subjected to full ophthalmic history taking and examination including LogMAR best-corrected visual acuity (BCVA), slit lamp of the anterior segment, applanation tonometry, and fundus slit lamp biomicroscopy. The diagnosis of macular hole was established both clinically and by optical coherence tomography (OCT). All cases were followed for at least 6 months anticipating spontaneous closure, which has been reported in similar cases. Seven cases were excluded due to spontaneous closure. Finally, 26 cases (22 males and 4 females) were included.

### 2.1. Surgical Method

After approval from the ethical committee, the surgical procedure was explained to all patients or their relatives, and written consent was signed. For children or adults who refused to adopt the strict face-down position, we used silicone oil as a tamponade (10 cases). In all other cases, 14% perfluoropropane (C_3_F_8_) was used. All surgeries were performed under general anaesthesia. Standard 23 G three-port sclerotomies were conducted, followed by vitrectomy with induction of posterior vitreous detachment if not already detached and fluid air exchange followed by ILM staining with blue dye. Then, the dye was washed away with fluid air exchange. The ILM was then peeled in a rhexis manner, with Eckardt end-gripping pick forceps. The aim was to peel at least 2 disc diameter areas of ILM 360° around the hole. Fluid air exchange was then performed.

For the gas-treated eyes, a 20 mL syringe containing 14% C_3_F_8_ gas was connected to an infusion cannula. The assistant injected 17 mL through the infusion cannula while the main surgeon allowed gas and air to escape from the superior temporal sclerotomy site via a flute needle. With this method, the vitreous cavity is filled with 14% C_3_F_8_. Sclerotomy ports were removed and their sites were tested for any leakage, which was closed with 7-0 Vicryl sutures. For young patients, all sclerotomy sites were sutured. If hyponony was encountered when eye tension was digitally tested at the end of the procedure, then the remaining 3 mL of gas was added to the tamponade by injection through the pars plana with a 30g needle.

For silicon-treated eyes, after fluid air exchange, one of the upper 2 sclerotomies was used to inject silicone while air is allowed to escape via a flute needle in the other sclerotomy.

Subconjunctival cefuroxime and dexamethasone were injected at the end of the procedure. Postoperatively, combined antibiotic-steroid drops (tobramycin 0.3% with dexamethasone 0.1%) were used 5 times daily for four weeks and atropine drops were applied three times a day for 2 weeks.

For gas-filled eyes, the patients were instructed to position their faces down until 50% of the gas was absorbed or for at least 2 weeks. For silicon-filled eyes, the patients were instructed to position their faces down as much as possible (at least 50% of daytime) for 2 weeks.

### 2.2. Follow-Up

**Figure 1:**
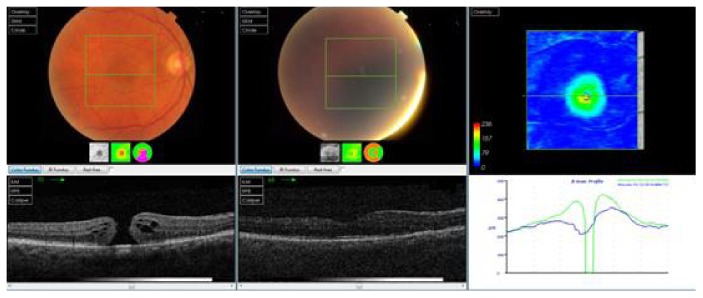
OCT scan of one of the cases in the silicone oil group (preoperative and 6 months postoperative).

**Figure 2:**
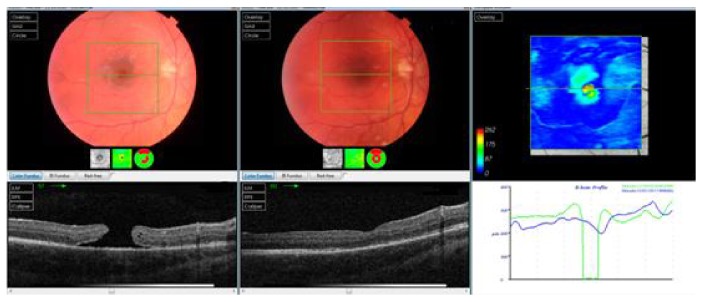
OCT scan of one of the cases in the C_3_F_8_ group (preoperative and 6 months postoperative).

Gas-filled eyes were followed up the day after the operation, after 1 week, and every month until the gas was absorbed; then, follow-up was conducted every 2 months for at least 1 year after the last surgery. Silicone oil-filled eyes were followed up the day after the operation, after 1 week, after 2 months, the day after silicone oil removal (4 months after the surgery), and every 2 months for at least 6 months after silicone oil removal. At every follow-up, visual acuity, intraocular pressure, slit lamp, and fundus exams were performed. OCT was performed every 2 months until the last follow-up, as shown in Figures 1 and 2.

The results were collected and statistically analysed.

## 3. Results

Thirty-three cases with traumatic macular hole were recruited during the study period. All cases were followed up for 6 months following the trauma in anticipation of spontaneous closure, which occurred in seven cases that were excluded from the study. The remaining 26 cases (22 males and 4 females) were reviewed, including 10 cases (38.4%) with silicone oil tamponade and 16 cases (61.6%) with 14% C_3_F_8_ gas tamponade. The age of the patients in the silicone oil group ranged from 9 to 43 years (mean 22.5 ± 12.7 years). The age of the patients in the C_3_F_8_ group ranged from 17 to 54 years (mean 30 ± 10 years). The mean preoperative LogMAR BCVA in the silicone oil group was 0.8 ± 0.4 (range 1.3 to 0.3). The mean preoperative BCVA in the C_3_F_8_ group was 1.1 ± 0.2 (range 1.3 to 0.7). The macular hole size in the silicone oil group (measured by Topcon OCT 2000) ranged from 289 *μ*m to 533 *μ*m with a mean of 404 ± 85. The macular hole size in the C_3_F_8_ group ranged from 354 *μ*m to 490 *μ*m with a mean of 401 ± 35.

### 3.1. Anatomical Success Rate

Twenty four (92.3%) cases achieved anatomical closure at 6 months while only 2 cases (7.4%) failed to close. Success rate was 90% in the silicone-filled (9/10) and 94% in the gas-filled (15/16) eyes with no statistically significant difference. Only one case in each group failed to close.

### 3.2. Visual Outcomes

The mean postoperative BCVA at one month in the silicone oil group was 0.5 ± 0.21 (range 1.0 to 0.3) compared to 0.4 ± 0.19 (range 1.0 to 0.3) in the C_3_F_8_ group. The mean postoperative BCVA at 4 months in the silicone oil group was 0.4 ± 0.22 (range 1.0 to 0.3) compared to 0.3 ± 0.13 (range 0.4 to 0.1) in the C_3_F_8_ group. The postoperative BCVA at 6 months in the silicone oil group ranged from 1.0 to 0.1 with a mean 0.3 ± 0.25, while in the C_3_F_8_ group the mean BCVA at 6 months was 0.2 ± 0.13 with a range from 0.5 to 0. The *t*-test was used to compare the preoperative BCVA at 6 months in the 2 groups. The two-tailed *p* value equals 0.0425; this difference is considered to be slightly statistically significant.

Table 1 shows the data from all eyes included in the study.

Confidence interval: The mean of group one minus group two equals −0.129, and the 95% confidence interval of this difference ranges from −0.253 to −0.005.

The intermediate values used in these calculations are as follows: *t* = 2.1424 and the standard error of difference = 0.060.

### 3.3. Adverse Events

Two cases (7.4%) had permanent nonclosure of the macular hole (one case in each group). Two cases in the gas-treated group had a postoperative day 1 high intraocular pressure that was medically controlled for 2 weeks. None of the cases in the study developed endophthalmitis, choroidal haemorrhage, or retinal detachment. The incidence of cataract was 33% (three of ten) for the silicone oil group and 25% (four of sixteen) in the gas-treated group.

## 4. Discussion

Thirty-three cases of TMH were reviewed in a retrospective comparative study done from May 2014 to September 2016. After the initial period of 6 months follow-up, 7 cases were excluded due to spontaneous closure. PPV + ILM peeling was performed in all cases. In 16 cases, 14% C_3_F_8_ was used as a tamponade. For children or adults refusing to adopt the strict facedown position, we used silicone oil as a tamponade (10 cases).

The results showed closure rate for gas-treated eyes (94%: 15 of 16) and closure rate for silicone oil tamponade (90%: 9 of 10) in a single operation. However, the slight difference in the percentage could be due to the different number of eyes included in the 2 studied groups. The primary success rate of traumatic macular holes closure was 92.3% (24 of 26).

The visual results showed that the postoperative LogMAR BCVA at six months in the silicone oil group ranged from 1.0 to 0.1 with a mean 0.3 ± 0.25, while the postoperative BCVA at six months in the C_3_F_8_ group ranged from 0.5 to 0 with a mean of 0.2 ± 0.13. Postoperative BCVA was compared at six months in the two groups by *t*-test. There was a statistically significant difference with better BCVA in the gas group. This might be due to the nature of the cases, like silicone oil, being used for larger traumatic macular holes with consequently more photoreceptor and RPE damage. Anatomical closure rates in this study were favorably compared with previous reports of traumatic macular hole 20 as well as myopic [20] or idiopathic hole [21]. This could be attributed to younger patient age, relatively earlier diagnosis, and the fact that overall natural closure rate of traumatic macular hole is higher than that of myopic or idiopathic hole [22–24].

For silicon-filled eyes, the final success rate (90%) is more than that reported by Goldbaum et al. [25] whose success rate was 83% seal rate for idiopathic macular holes, and this may be due to the fact that they operated on their cases and did not instruct the patients to adopt special position in the early postoperative period, and considerably less than the 97% reported previously by Pertile and Claes [26] for idiopathic macular holes.

In this study, the anatomical closure rates for gas-filled eyes (94%) compared to the 58% closure rate were first described by Kelly and Wendel [27] and the 69% seal rate described by Freeman et al. [28]. This might be because the macular holes in these studies were idiopathic and not traumatic; additionally, younger patients were included in our study with subsequent healthy RPE and ILM peeling was performed in our study.

We also analyzed pre- and postoperative visual acuities. The average preoperative visual acuity was slightly worse in the silicone oil group compared with the gas group. Both groups showed a gradual improvement in LogMAR visual acuities at four weeks, four months, and six months with the silicone oil group improved from 0.8 to 0.3, while the gas-treated group improved from 1.1 to 0.2. Goldbaum et al. [25] reported anatomical closure of idiopathic macular holes with silicone oil which resulted in an improvement of 3-4 lines; however, this result was not reported for traumatic macular holes. The better visual outcome in gas-treated eyes than in silicone-treated eyes may be due to large hole size with consequently more photoreceptor and RPE damage and the potential toxicity of silicone oil when in contact with the bare RPE and photoreceptors [29–32].

## 5. Conclusions

Spontaneous closure of TMH could occur in a significant percentage of cases, so an initial period of follow-up is advised. In cases treated with PPV with ILM peeling, both silicone oil and C_3_F_8_ can achieve comparable anatomical and functional results.

## Figures and Tables

**Table 1:** Shows the data from all patients.

Case	Sex	Age	Pre-BCVA	Macular hole size	BCVA one month	BCVA 4 months	OCT 4 months	BCVA 6 months	OCT 6 months
Silicone									
1	M	19	0.7	378	0.4	0.3	Hole closed	0.3	Hole closed
2	M	14	0.5	289	0.4	0.3	Hole closed	0.1	Hole closed
3	M	43	1.0	478	0.3	0.3	Hole closed	0.2	Hole closed
4	M	26	1.3	503	0.7	0.5	Hole closed	0.4	Hole closed
5	M	16	0.5	291	0.5	0.4	Hole closed	0.3	Hole closed
6	F	32	1.3	415	0.4	0.3	Hole closed	0.2	Hole closed
7	M	14	0.3	433	0.4	0.3	Hole closed	0.3	Hole closed
8	M	10	1.0	390	0.3	0.3	Hole closed	0.3	Hole closed
9	M	9	1.3	330	0.4	0.3	Hole closed	0.3	Hole closed
10	F	42	1.3	533	1.0	1.0	Failure	1.0	Failure
	Mean	22.5	0.8	404	0.5	0.4		0.3	
	MAX	43	0.3	533	0.3	0.3		0.1	
	Min	9	1.3	289	1.0	1.0		1.0	
	SD	12.7	0.4	85.13	0.21	0.22		0.25	
C_3_F_8_									
1	M	17	0.7	390	0.3	0.3	Hole closed	0.1	Hole closed
2	F	23	1.0	394	0.3	0.3	Hole closed	0.1	Hole closed
3	M	36	1.3	443	0.4	0.1	Hole closed	0	Hole closed
4	M	32	1.0	422	0.5	0.4	Hole closed	0.3	Hole closed
5	M	25	1.0	354	0.5	0.1	Hole closed	0.1	Hole closed
6	M	20	1.0	406	1.0	0.3	Failure	0.5	Failure
7	M	18	0.8	389	0.7	0.4	Hole closed	0.2	Hole closed
8	M	54	1.0	402	0.4	0.1	Hole closed	0.1	Hole closed
9	M	33	1.0	403	0.3	0.3	Hole closed	0.1	Hole closed
10	M	30	1.3	399	0.4	0.1	Hole closed	0.1	Hole closed
11	F	21	1.3	401	0.3	0.1	Hole closed	0.3	Hole closed
12	M	34	1.0	432	0.3	0.1	Hole closed	0.1	Hole closed
13	M	38	1.3	490	0.4	0.4	Hole closed	0.3	Hole closed
14	M	43	1.3	389	0.5	0.3	Hole closed	0.3	Hole closed
15	M	32	1.3	354	0.5	0.4	Hole closed	0.3	Hole closed
16	M	23	1.0	355	0.5	0.5	Hole closed	0.1	Hole closed
	Mean	29.94	1.1	401.44	0.4	0.3		0.2	
	MAX	54	0.7	490	0.3	0.1		0	
	Min	17	1.3	354	1.0	0.4		0.5	
	SD	9.98	0.2	34.8	0.19	0.13		0.13	
